# Prevalence of hyposalivation and associated factors in survivors of head and neck cancer treated with radiotherapy

**DOI:** 10.1590/1678-7757-2020-0854

**Published:** 2021-04-19

**Authors:** Riéli Elis Schulz, Laura Izabel Lampert Bonzanini, Gabriela Barbieri Ortigara, Eloisa Barbieri Soldera, Cristiane Cademartori Danesi, Raquel Pippi Antoniazzi, Kívia Linhares Ferrazzo

**Affiliations:** 1 Universidade Federal de Santa Maria Santa MariaRS Brasil Universidade Federal de Santa Maria, Santa Maria, RS, Brasil.; 2 Universidade Federal de Santa Maria Faculdade de Odontologia Departamento de Patologia Santa MariaRS Brasil Universidade Federal de Santa Maria, Faculdade de Odontologia, Departamento de Patologia, Santa Maria, RS, Brasil.; 3 Universidade Federal de Santa Maria Faculdade de Odontologia Departamento de Estomatologia Santa MariaRS Brasil Universidade Federal de Santa Maria, Faculdade de Odontologia, Departamento de Estomatologia, Santa Maria, RS, Brasil.

**Keywords:** Head and neck cancer, Radiotherapy, Xerostomia

## Abstract

Hyposalivation and sensation of dry mouth (xerostomia) are one of the most common adverse effects in the treatment of patients with head and neck cancer.

**Objective:**

This study evaluates the prevalence of late hyposalivation and associated factors in survivors of squamous cell carcinoma of the oral cavity, oropharynx, hypopharynx, or larynx treated with radiotherapy with or without concomitant chemotherapy.

**Methodology:**

A cross-sectional study was conducted with 88 patients who had concluded radiotherapy at least three months before the study, at a referral center for the treatment of head and neck cancer in the Southern region of Brazil. Hyposalivation was evaluated based on the stimulated salivary flow rate using the spitting method. Multivariate analysis using binary logistic regression was performed to determine the associations between hyposalivation and clinical and demographic variables.

**Results:**

Hyposalivation was found in 78.41% of the sample and the mean radiation dose was 63.01 Gy (±9.58). In the crude model of the multivariate analysis, hyposalivation was associated with higher doses of radiation (p=0.038), treatment with concomitant radiotherapy and chemotherapy (p=0.005), and time elapsed since the end of radiotherapy (p=0.025). In the adjusted model of the multivariate analysis, hyposalivation was only associated with dose and time elapsed. Patient who received higher doses of radiation had a 4.25-fold greater chance of presenting hyposalivation, whereas a longer time elapsed since the end of radiotherapy exerted a 75% protective effect against the occurrence of hyposalivation.

**Conclusion:**

Hyposalivation is a highly prevalence late-onset side effect of radiotherapy in patients treated for head and neck cancer, with a greater chance of occurrence among those who received higher doses of radiation and those who ended therapy less than 22 months before our study. Concomitant chemotherapy and radiotherapy does not seem to increase the chances of hyposalivation compared to radiotherapy alone.

## Introduction

Cancer of the oral cavity, pharynx, and larynx corresponds to approximately 4.6% of all cases of cancer worldwide.^1^ In 2018, 834,860 new cases of cancer in these anatomic sites were diagnosed.^1^ When anatomic sub-sites are analyzed, the incidence of cancer of the mouth and oropharnyx is higher, followed by cancer of the larynx and hypopharynx.^2^ Due to demographic changes, the number of cases of lip, mouth, and pharyngeal cancer is expected to increase by 62%, reaching 856,000 cases annually by 2035.

Radiotherapy (RT) is widely employed in the treatment of head and neck cancer used as primary therapy, adjunct therapy to surgery, with concomitant chemotherapy (CT) or as palliative treatment. High doses of RT can negatively affect the soft and hard tissues of the oral cavity.^3^ Patients with head and neck cancer are generally irradiated with high doses (50 to 70 Gy),^4^ which side-effects include hyposalivation, trismus, and dysphagia; in hard tissues, the effects may be osteoradionecrosis and radiation caries – all of which have a late-onset.^3^

One of the main problems resulting from radiotherapy in the head and neck region is the damage to glandular tissues, reducing the salivary flow.^5^ Hyposalivation occurs due to cell death and fibrosis of the glandular tissue caused by radiotherapy, leading to a sensation of dry mouth (xerostomia).^6^ Some studies suggest a significant increase in late-onset xerostomia in patients treated with concomitant chemotherapy and radiotherapy,^7^ whereas other authors state that there is no strong evidence of the additive effect of chemotherapy concomitant to radiotherapy on hypofunction of the salivary glands.^8^ In a systematic review, Jensen, et al.^8^ (2010) found divergences in the literature regarding hyposalivation and xerostomia. According to some authors, the term xerostomia is often used as a synonym of hyposalivation, when it should only be used to indicate the perception of dry mouth reported by the patient.

Due to this common late effect in patients irradiated in head and neck and the influence on their quality of life, studies bringing new information to the field are necessary. Therefore, this study evaluates the prevalence and factors associated with late hyposalivation based on the stimulated salivary flow rate in patients treated with 3D conformal radiotherapy with or without concomitant chemotherapy for the treatment of cancer of the oral cavity, oropharynx, hypopharynx, or larynx. We hypothesize that a high prevalence would be found caused by high doses of radiation in the salivary glands and also influenced by the type of therapy used in this region in Southern Brazil.

## Methodology

### Study design, sample and eligibility criteria

An observational, quantitative, analytical, cross-sectional study was conducted. Data were collected from April 2016 to May 2017 at the Head and Neck Surgery outpatient clinic of the Federal University of Santa Maria hospital, which is the largest public hospital in the central region of the state of Rio Grande do Sul, Brazil, and a reference for the treatment of head and neck cancer. Survivors of cancer of the oral cavity, oropharynx, hypopharynx, or larynx who had undergone 3D conformal radiotherapy and had completed treatment at least three months prior to the process of data collection were screened for eligibility. The inclusion criteria were men or women aged 18 years or older, presumably disease free who agreed to undergo the proposed examinations.

The sample size was calculated based on the difference in mean non-stimulated salivary flow between individuals exposed to a radiation dose of 50 Gy and non-exposed individuals in a previous study: 0.47 (SD: 0.31) and 0.28 (SD: 0.32), respectively.^9^ Considering a 5% significance level and 80% power, a minimum of 59 participants was needed. Due to the multivariate analysis and the possibility of dropouts, considering the participants’ vulnerability, the sample size was increased by 30% (minimum of 85 participants).

This study was approved by the Human Research Ethics Committee of the Federal University of Santa Maria (certificate number: 1.387.994/2016). All participants signed an informed consent form.

### Data collection

Demographic characteristics (age, sex, and ethnicity), lifestyle habits (smoking and alcohol use), comorbidities (defined as any condition able to modify salivary flow, as diabetes mellitus, Sjogren syndrome, or hypothyroidism, and/or use of drugs that can induce salivary gland hypofunction and/or xerostomia, as antihypertensive agents, antidepressant and others)^10^^,^^11^ as well as data regarding the disease (tumor type and stage) and treatment (type and dose of radiation) were collected from the patient medical charts. Xerostomia (subjective assessment) was recorded based on the answer to the following question: “Does your mouth generally feel dry?”^12^^,^^13^

### Saliva collection and sialometry - stimulated salivary flow

The participants were instructed not to eat, drink (except water) or smoke at least one hour prior to the saliva collection. Stimulated salivary flow rate was determined using the spitting collection method.^14^ The mechanical stimulation of salivation was performed with a sterile rubber strip with a standardized size (2ϗ2 cm). The collection lasted five minutes. All saliva collection procedures were held between 13:30 and 3:30 pm. The participant was seated comfortably on a chair and were instructed neither to speak nor interrupt the data collection process; otherwise, a new collection would be initiated. The saliva from the first minute was discarded to eliminate possible food scraps that could influence the weight of the saliva. Then, the participant expelled saliva into a previously sterilized and weighed universal collector at 60-second intervals. The collection time was controlled with a chronometer. The total quantity of stimulated saliva was determined based on weight measured using a precision scale (Balança Eletrônica Gehaka BG 200) expressed in grams. The total weight was divided by four (because the first minute was discarded) to obtain the salivary flow rate in grams per minute, which is similar to mL/min. Hyposalivation was recorded if the stimulated salivary flow rate was less than 0.5 mL/min.^15^

### Radiation caries

Ring-shaped caries on the cervical third of the vestibular, incisal, occlusal, and lingual faces of the teeth were considered radiation caries,^4^ which were detected through a visual clinical examination aided by a wooden tongue depressor with the patient lying on the dental chair. The clinical examinations were performed by two raters who had previously undergone training and calibration exercises. The calibration involved the examination of 20 images displayed on a computer screen one at a time, for which the raters marked “yes” or “no” on a chart. The procedure was repeated after 30 days. The Kappa coefficient was estimated for the determination of intra-rater and inter-rater agreement (K=0.79 to 1.00).

### Statistical analysis

The data were analyzed descriptively, with the calculation of mean, standard deviation, and median values. The Kolmogorov-Smirnov and Shapiro-Wilk tests were used to determine the normality of the variables distribution. For the purposes of statistical analysis, age (62 years), time elapsed since the end of RT (22 months), and total radiation dose (66 Gy) were dichotomized by the median. Other variables were also dichotomized for statistical purposes. Tumor location was dichotomized as mouth/oropharynx or hypopharync/larynx. Tumor stage was classified as AJCC staging system 7^th^ edition^16^ and it was dichotomized as initial (stages I and II) or advanced (stages III and IV). Type of treatment was dichotomized as RT without CT or RT with CT. Stimulated salivary flow and xerostomia were compared between patients with and without hyposalivation using Fischer's exact test and the Mann-Whitney test, with 5% significance level.

Binary logistic regression models were run to evaluate associations between hyposalivation and covariables. The following categorical variables were evaluated in the crude model: age, tumor location, stage, type of treatment, time elapsed since the end of treatment, radiation doses and comorbidities/medications that cause xerostomia. Variables with high p-value were removed from the model and only those with a p-value <0.20 were incorporated into the adjusted model. Data were analyzed using the Statistical Package for the Social Sciences (SPSS, version 21.0, PASW, Chicago, IL, USA).

## Results

The response rate was 89.79% (88/98). Ten cases were excluded because participants were unable to remain until the completion of all examinations due to public transportation schedule. Thus, the sample was composed of 73 men and 15 women (mean age: 62.74±9.70 years) with malignant tumors of the mouth/oropharynx (59.51%), larynx (35.2%), and hypopharynx (5.7%). Most cases were in the advanced stage (68.2%). Table 1 shows the complete description of the sample.

**Table 1:** Distribution of demographic and clinical variables in groups with and without hyposalivation

	Without Hyposalivation	With Hyposalivation
	n (%)	n (%)
**With Hyposalivation**		
≤ 62 (median)	10 (52.6)	39 (56.5)
> 62	9 (47.4)	30 (43.5)
**Sex**		
Female	2 (10.5)	13 (18.8)
Male	17 (89.5)	56 (81.2)
**Skin color**		
White	15 (78.9)	62 (89.9)
Non-white	4 (21.1)	7 (10.1)
Location*		
Mouth/oropharynx	8 (42.1)	44 (63.8)
Hypopharynx	1 (5.3)	4 (5.8)
Larynx	10 (52.6)	21 (30.4)
Stage*		
I	4 (21.1)	11 (15.9)
II	1 (5.3)	12 (17.4)
III	10 (52.6)	15 (21.7)
IV	4 (21.1)	31 (44.9)
Type of treatment*		
Radiotherapy	2 (10.5)	4 (5.8)
Surgery + Radiotherapy	8 (42.1)	9 (13.0)
Surgery + Radiotherapy + Chemotherapy	7 (36.8)	26 (37.7)
Radiotherapy + Chemotherapy	2 (10.5)	30 (43.5)
Time elapsed since radiotherapy*		
≤ 22 months (median)	5 (26.3)	39 (56.5)
> 22 months	14 (73.7)	30 (43.5)
Dose (Gy*)		
≤ 66 Gy (median)	14 (77.8)	33 (49.3)
> 66 Gy	4 (22.2)	34 (50.7)
**Dentition**		
Normal	1 (5.3)	6 (8.7)
Partially edentulous	13 (68.4)	47 (68.1)
Edentulous	5 (26.3)	16 (23.2)
**Radiation caries**		
No	9 (64.3)	36 (67.9)
Yes	5 (35.7)	17 (32.1)
**Comorbidities/xerostomic drugs**		
No	12 (63.2)	30 (43.5)
Yes	7 (36.8)	39 (56.5)

* dichotomized for statistical analysis; a chi-squared test; b Fisher's exact test

Hyposalivation was found in 69 individuals (78.41%), among whom mean stimulated salivary flow was 0.21 mL/min (±0.16). Mean stimulated salivary flow among the patients without hyposalivation was 1.25 mL/min (p<0.001). Xerostomia was reported by 82 participants (93.2%). An association was found between the perception of xerostomia and the objective assessment of salivary flow (p=0.018) (Table 2).

**Table 2:** Stimulated salivary flow rate (mL/min) and perception of xerostomia in patients with and without hyposalivation

	Total	Without hyposalivation	With hyposalivation	p
		(n = 19)	(n = 69)	
SSF* **(mL/min)**				
Mean ± standard deviation	0.43 (±0.62)	1.25 (± 0.94)	0.21 (± 0.16)	<0.001^a^
Median (P25-P75)		0.88 (0.64 – 1.39)	0.22 (0.03 – 0.36)	
**Xerostomia**				
No	6 (6.8%)	4 (21.1%)	2 (2.9%)	0.018^b^
Yes	82 (93.2%)	15 (78.9%)	67 (97.1%)	

* SSF: stimulated salivary flow; a Mann-Whitney test; b Fisher's exact test

The patients received different cancer treatment modalities: RT alone (6.8%), surgery + RT (19.3%), surgery + RT + CT (37.5%) and RT + CT (36.4%). In the crude analysis, an association was found between type of treatment and hyposalivation. Patients who had been treated with RT + CT had a 4.79-fold greater chance of presenting hyposalivation than those who had not been treated with concomitant chemotherapy (p=0.005). Radiation dose and time elapsed since the end of radiotherapy were also associated with hyposalivation in the crude analysis. Mean radiation dose was 63.01 Gy (±9.58) and higher doses were associated with a greater chance of exhibiting hyposalivation (p=0.038). Time elapsed since the end of the radiotherapy ranged from three to 192 months. A shorter time since the end of treatment was associated with a greater chance of hyposalivation (p=0.025). After the adjustment for possible confounding variables, time since the end of treatment (p=0.022), and radiation dose (p=0.024) remained significantly associated with the outcome. Patients who had received higher doses of radiation had a 4.25-fold greater chance of having hyposalivation. A longer time elapsed since the end of treatment exerted a protective effect, denoting a 75% lower chance of hyposalivation (Table 3). Hyposalivation was not associated with age, sex, location of primary tumor, or radiation caries.

**Table 3:** Associations between hyposalivation and demographic, behavioral and clinical variables

	Hyposalivation	OR (95% CI)	p*	OR (95% CI)	p**
	N (%)	Crude		Adjusted	
**Location**				–	–
Mouth/oropharynx	44 (63.8)	1	0,094		
Hypopharynx and larynx	25 (36.2)	0.41 (0.15 – 1.16)			
**Chemotherapy**					
No	13 (18.8)	1	0,005	–	–
Yes	56 (81.2)	4.79 (1.62 – 14.15)			
**Dose (Gy)**					
≤ 66	33 (47.8)	1	0,038	1	0,024
> 66	34 (49.3)	3.61 (1.08 – 12.09)		4.25 (1.21 – 14.94)	
**Time since radiotherapy**					
≤ 22 months	39 (56.5)	1	0,025	1	0,022
> 22 months	30 (43.5)	0.28 (0.09 – 0.85)		0.25 (0.08 – 0.82)	
**Comorbidities/ drugs that cause xerostomia**					
No	30 (43.5)	1	0,133	–	–
Yes	39 (56.5)	2.23 (0.78 – 6.35)			

* Crude and adjusted

** binary logistic regression; OR: odds ratio

(-): variables not retained in final model

Variables analyzed: age, tumor location, stage, time elapsed since the end of treatment, radiation dose, type of treatment and comorbidities/xerostomic drugs; Only variables with p-value < 0.20 in the crude model were incorporated into the adjusted model.

**Figure 1:**
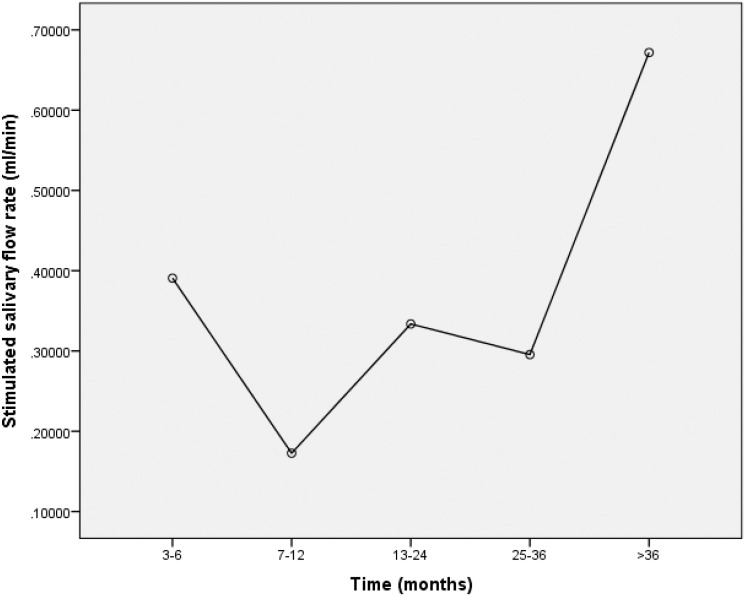
Mean stimulated salivary flow as function of time elapsed since radiotherapy, in the total sample

**Figure 2:**
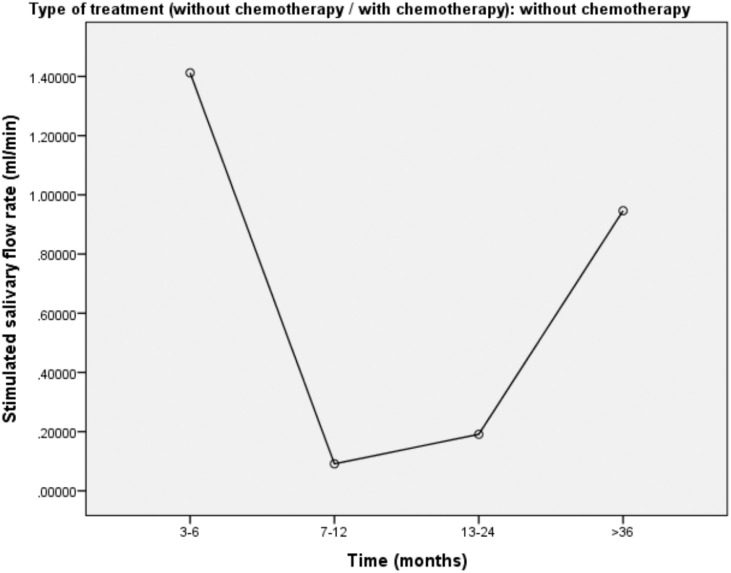
Mean stimulated salivary flow according to time in patients treated with radiotherapy

**Figure 3:**
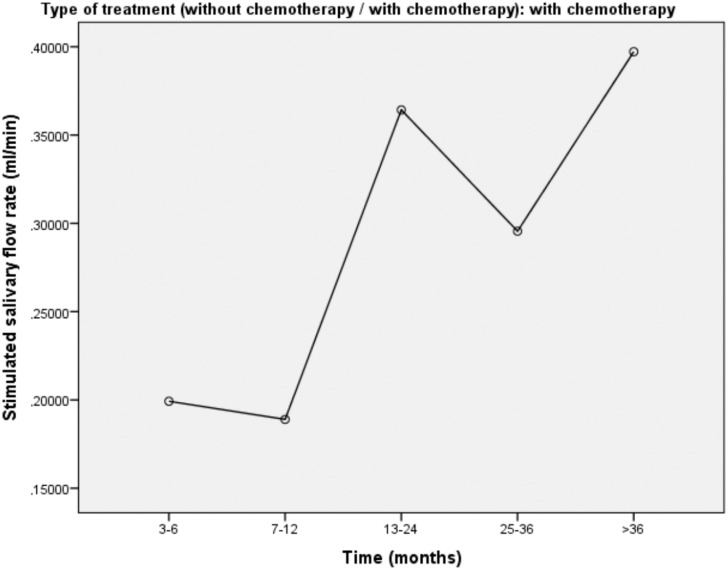
Mean stimulated salivary flow according to time in patients treated with radiotherapy plus chemotherapy

Figures 1, 2, and 3 show the mean rate of stimulated salivary flow as a function of the time elapsed since radiotherapy in total sample, in patients treated with radiotherapy and in patients treated with radiotherapy plus chemotherapy, respectively.

## Discussion

In this study, hyposalivation and associated factors were evaluated in patients who underwent radiotherapy of the head and neck. The prevalence of hyposalivation was high and it was associated with both higher doses of radiation and shorter time since the end of radiotherapy treatment. Previous studies have demonstrated that the adverse effects on the soft tissues (particularly the salivary glands) and hard tissues of the oral cavity are directly proportional to the radiation dose and the type of radiotherapy.^17^^,^^18^

Some studies offer information on threshold doses of radiation to avoid harm to the salivary glands and consequent occurrence of hyposalivation. Marks, et al.^19^ (1981) found that a dose from 30 to 40 Gy was able to cause an accentuated reduction in salivary flow. Moretto, Rampino, and Munoz^20^ (2014) demonstrated that 32 Gy is the average dose to spare the parotid gland from harm. Blanco, et al.^18^ (2005) estimated that a mean dose of 25.8 Gy reduced the flow from a single parotid gland to 25% of its pre-treatment amount, regardless of the radiotherapy method. Therefore, the fact that the patients in this study received a mean radiation dose of 63.01 Gy explains the high prevalence of hyposalivation in the sample.

Another significant finding of this investigation was the protective effect of the time elapsed since the end of radiotherapy on salivary flow. A longer time since the end of the therapy was associated with a lower chance of presenting hyposalivation. Despite the cross-sectional design of this study, simulated salivary flow was greater in those who had finished treatment a longer period of time before the evaluation, especially 36 months earlier. Previous studies have also demonstrated salivary flow recovery over time. Eisbruch, et al.^21^ (1999) found a 78.4% reduction in stimulated salivary flow one month after irradiation compared to the pre-radiation period; one year after radiotherapy, median stimulated salivary flow was 114% of pre-radiation amount, suggesting the complete recovery of saliva production. The authors also found that the salivary flow recovery from the parotid gland occurred with doses up to 25 to 30 Gy, suggesting that the recovery of glandular tissues and the consequent improvement in salivary flow depends on the radiation dose.

Jensen, et al.^8^ (2010) found that non-stimulated and stimulated salivary flow rates were lower during radiotherapy and diminished further from one to three months after the treatment. The authors also found a slight increase in stimulated salivary flow six months after treatment and in non-stimulated salivary flow one year after treatment. Other authors state that salivary gland recovery occurs, on average, within two years after the completion of radiotherapy.^22^^,^^23^

Radiotherapy with concomitant chemotherapy is the protocol of treatment for patients with epidermoid carcinoma of the head and neck in advanced stages whether as definitive treatment for the preservation of the organ or indicated for patients at high-risk in the postoperative period.^24^ In our study, a strong association was found between RT + CT and the reduction in stimulated salivary flow in the crude analysis, but this association was lost after controlling for confounding variables in the adjusted multivariate analysis.

Some studies have found a statistically significant association between RT + CT and the subjective symptom of xerostomia,^7^^,^^25^ but the authors did not assess salivary flow. Hey, et al.^26^ (2009) reported lower doses of tolerance for radiotherapy with concomitant chemotherapy in patients who received cisplatin as the chemotherapeutic drug. The dose tolerated by patients who received RT + CT was at least 7 to 8 Gy lower that that tolerated by patients who received radiotherapy alone. These findings demonstrate a greater tendency toward damage to the tissue of the parotid gland caused by radiotherapy with concomitant chemotherapy. However, other authors state that no concrete conclusion can be drawn regarding the additive effect of chemotherapy associated with radiotherapy on harm to the salivary glands leading to hyposalivation.^8^ According to Chao, et al.^27^ (2001), neither the treatment modality (with or without chemotherapy) nor the radiation technique (intensity-modulated radiation therapy [IMRT] or non-IMRT) exert an independent influence on the functional outcome for the salivary glands; only the dose exerts such an influence. Few studies have examined the long-term effect of chemotherapy. Meurman, et al.^28^ (1997) followed up patients with lymphoma for up to five years after chemotherapy and found no changes in stimulated or non-stimulated salivary flow.

The divergences in the findings reported in the literature may be due to the fact that some studies only compared xerostomia with the type of treatment (without measuring salivary flow) or due to the type of analysis performed. Some studies report that the evaluation of xerostomia alone is not a secure indicator of a reduction in saliva production.^13^ This may be explained by the change in the composition of saliva caused by chemotherapy.^29^ In this study, an association was found between xerostomia and hyposalivation, as patients who reported a sensation of dry mouth had a low stimulated salivary flow rate. Nonetheless, no association was found between RT + CT and hyposalivation in the adjusted multivariate analysis, despite the fact that the crude analysis suggested such association.

In the literature, the minimum cutoff point for total stimulated salivary flow ranges from 0.5 to 0.7 mL/min. In our study, hyposalivation was defined as a stimulated salivary flow rate of less than 0.5 mL/min, as suggested by Sreebny^15^ (2000). Considering the participants’ mean age and the fact that more than half of the sample had comorbidities and/or used medications that could reduce salivary flow and, mainly, it is a sample composed of patients who had their head and neck irradiated, we found it more prudent to use the criterion that represented a more significant reduction in the total volume of stimulated salivary flow. Regarding xerostomia, different from other authors,^30^ we used a tool easier to apply in order to assess this variable,^12^^,^^13^ once the main purpose of the study was the objective assessment of hyposalivation.

This investigation has limitations that should be addressed. It was a cross-sectional study and no evaluations of salivary flow or xerostomia were performed in the pre-radiotherapy period. It was also not possible to measure the average radiation dose received in isolation by the parotid gland. Moreover, we did not exclude individuals who took medications that cause xerostomia, such as anti-hypertensive agents or antidepressants, or those participants who had some comorbidity that could cause xerostomia, such as diabetes. However, this possible bias was minimized by the inclusion of these independent variables in the statistical analysis. Comorbidities and xerostomic drugs that could reduce salivary flow had no statistically significant association with hyposalivation in both crude analysis or multivariate analysis adjusted for confounding variables.

Studies have demonstrated that IMRT produces less toxicity and fewer adverse effects compared to 3D conformal radiotherapy.^31^^,^^32^ All individuals in the study had been submitted to the latter form of radiotherapy, which may explain the findings. Despite the known benefits of IMRT, 3D-conformal radiotherapy continues to be widely employed in Brazil – particularly at public health services –, similar to what occurs in other countries, where patients do not have the opportunity to benefit from newer techniques due to the financial restrictions of public hospitals.

In brief, this study offers significant results that contribute to knowledge regarding hyposalivation and associated factors at a reference hospital in head and neck cancer treatment in Southern region of Brazil. This research will provide new information to the literature, drawing the attention of dentists and health professionals to this late adverse effect of radiotherapy, consequently leading to an improvement in prevention and treatment of these head and neck cancer patients. The results can be extrapolated to populations with similar conditions.

## Conclusion

Hyposalivation is a significant late-onset side effect of radiotherapy, with a high prevalence rate among patients submitted to irradiation of the head and neck region. This condition is also dose dependent. Chemotherapy concomitant to radiotherapy does not seem to increase the chances of hyposalivation compared to radiotherapy alone. A better understanding of the causes and factors that expose patients to a greater chance of having hyposalivation is essential to the development of preventive strategies and support therapies that can minimize the harm caused to patients.
